# Supplementary material for: Structured proxy features for multimodal NSCLC survival prediction from pretreatment CT
Source: Front Artif Intell. 2026 Aug 28;9:1865458. doi: 10.3389/frai.2026.1865458 (PMC13562299; doi:10.3389/frai.2026.1865458)
Supplement: Supplementary file 1 [file Supplementary_file_1.docx]

**Supplementary Material**

**Structured proxy features for multimodal NSCLC survival prediction from pretreatment CT**

Huu Phong Nguyen^1^, Delower Hossain^2^, Ehsan Saghapour^3^, Zhandos Sembay^3^, Jake Y. Chen^1,2,3,*^

1 Department of Biomedical Informatics and Data Science, School of Medicine, The University of Alabama at Birmingham (UAB), Birmingham, AL, United States

2 Department of Computer Science, The University of Alabama at Birmingham (UAB), Birmingham, AL, United States

3 System Pharmacology and AI Research Center (SPARC), The University of Alabama at Birmingham (UAB), Birmingham, AL, United States

*Corresponding author: Jake Y. Chen, jakechen@uab.edu

# Implementation Details

This supplementary material provides the full implementation details for each experiment described in Section 4. Settings that are shared across all or most experiments are consolidated in Section S1 to avoid repetition; individual experiment sections (S2–S6) describe only the aspects that are specific to each experiment.

## S1. Common Experimental Settings

Model parameters and notation are summarized in Table 1; key hyperparameters across all components are listed in Table 2.

### S1.1 Data Partitioning

All experiments used the same fixed cohort split: patients were partitioned into a training set (2/3) and a held-out test set (1/3) using a stratified split by event status (random seed fixed at 42). All feature selection and model training was performed exclusively on the training partition.

### S1.2 Modality Arms

Four modality arms were used throughout all multimodal experiments:

- **Clinical (C):** Seven encoded covariates – age, sex, T-stage, N-stage, M-stage, overall stage, and histology.
- **Radiomics (R):** 1,688 normalized first- and higher-order imaging features derived from the tumor region.
- **TMAE:** 768-dimensional latent representations extracted from the [CLS] token of the self-supervised Vision Transformer encoder.
- **Simulation (SM):** Six parameters – tumor growth rate, necrosis ratio, proliferation coefficient α, necrosis coefficient β, radiomic entropy, and radiomic sphericity.

### S1.3 Survival Models

Three survival models were evaluated in all experiments:

- **Cox Proportional Hazards (CoxPH):** Fitted with LASSO penalization (λ = 0.1).
- **Neural Cox:** A feedforward neural survival model (see architecture below).
- **Ensemble:** Equal-weight combination of CoxPH and Neural Cox risk scores (weight = 0.5 each).

### S1.4 Neural Cox Architecture

The Neural Cox model comprised two fully-connected hidden layers of dimensions [32, 16] with batch normalization, ReLU activations, and dropout (rate = 0.30). Training used the Adam optimizer (learning rate = 0.001, weight decay = 10^−3^, batch size = 16) for up to 200 epochs, with early stopping (patience = 20) monitored on a 15% internal validation partition held out from the training set.

### S1.5 Performance Metrics

All experiments reported the following metrics on the held-out test set: concordance index (C-index) (Uno et al., 2011), integrated time-dependent AUC (iAUC) (Heagerty et al., 2000), log-rank p-value, and Cox-derived hazard ratio (HR) between median-split high- and low-risk groups.

### S1.6 TMAE Feature Selection Pipeline

Where TMAE feature selection was applied, a two-stage cross-validated pipeline was used exclusively on the training partition: (1) pairwise Pearson correlation filtering removing features with |r| above a threshold ρ; (2) 10-fold cross-validation frequency filtering retaining only features selected in more than a threshold proportion τ of folds. The thresholds ρ and τ were either optimized (Bayesian search experiments) or swept (ablation experiments), as described in the individual sections.

### S1.7 Simulation Model Configuration

The Simulation (SM) model is a 3D cellular automaton operating on a 90³ voxel grid over 120 iterations. The proliferation and necrosis parameters are derived from radiomics as α = a_0_ + a_1_·E and β = b_0_ + b_1_·(1 − S), where E is radiomic entropy and S is sphericity, consistent with Equation (5). Simulation outputs, specifically tumor growth rate (net change in viable cell count per iteration) and necrosis ratio (fraction of necrotic cells at simulation end), are propagated as SM features into the downstream fusion model.

## S2. Comparative Evaluation of Imaging Models (Section 4.2)

Experiment-specific settings. Two self-supervised autoencoders and one pretrained CNN were compared within the same multimodal fusion framework, each producing a 768-dimensional output: (1) TMAE – a Vision Transformer trained via masked patch reconstruction (self-supervised), representation from the [CLS] token; (2) CAE – a Convolutional Autoencoder with a hierarchical strided-convolution encoder and fully-connected bottleneck; (3) RN18 – a 3D ResNet-18 pretrained on 23 medical imaging datasets, used as a fixed feature extractor with a linear projection head mapping 512-dimensional global average-pooled output to 768 dimensions (no task-specific fine-tuning). The five-arm progressive ablation described in Section 4.2 was applied to each encoder, with TMAE feature selection stabilized via a sweep over ρ ∈ {0.60, 0.70, 0.80, 0.90} × τ ∈ {0.50, 0.60, 0.70, 0.80} and the threshold maximizing Neural Cox C-index selected for final reporting.

## S3. Sensitivity Analysis of Simulation Parameters (Section 4.4.2)

Experiment-specific settings. Twelve perturbation scenarios were evaluated by applying independent ±10% and ±20% relative changes to the proliferation coefficient α, the necrosis coefficient β, and both simultaneously. For each scenario, the Simulation model (Section S1.7) was re-executed from scratch for all test patients using the perturbed parameter values; patients for whom CT data could not be loaded retained their original simulation outputs. CT volumes were pre-loaded in parallel using multithreaded joblib workers. Feature selection was fixed at the optimal configuration from Section 4.4: TMAE thresholds ρ = 0.6162, τ = 0.5361 over 10 folds; top 75 Radiomics features by univariate Cox ranking; top 67.51% of Clinical features by univariate Cox p-value; all six SM features retained without filtering. The survival model was CoxPH only (LASSO λ = 0.1), trained on the fixed 2/3 training partition.

## S4. Bayesian Feature Selection and Model Optimization (Section 4.4.1)

Experiment-specific settings. Bayesian optimization was performed using the Tree-structured Parzen Estimator sampler with 700 total trials and 140 random startup trials. The objective function returned the maximum C-index achieved across the three survival models per trial. Five hyperparameters were jointly optimized: the TMAE correlation threshold ρ ∈ [0.60, 0.90], the TMAE selection frequency threshold τ ∈ [0.50, 0.80], the number of top Radiomics features k ∈ {25, 50, 75, 100} (ranked by univariate Cox p-value), the SM feature retention fraction πSM ∈ [0.0, 1.0], and the Clinical feature retention fraction πC ∈ [0.0, 1.0]. Feature selection was executed once per trial on the training partition and the resulting feature matrix passed to all three survival models on the same held-out test set.

## S5. Multimodal Feature Fusion Ablation Study (Section 4.3)

Experiment-specific settings. A total of 18 configurations were evaluated: four single-domain baselines, seven feature-level fusion combinations (concatenated features prior to model training), and seven signature-level fusion combinations (per-domain CoxPH risk scores concatenated as second-stage inputs). For the TMAE and Radiomics arms, feature selection followed the common two-stage pipeline (Section S1.6) with fixed thresholds: Pearson |r| > 0.80 for correlation filtering, and 70% cross-validation selection frequency (10-fold LASSO Cox filter). Radiomics features were further reduced to the top 10 by univariate Cox p-value; TMAE to the top 15. Clinical and SM features were used without cross-validation-based filtering.

## S6. Optimization of Simulation Coefficients for the Proposed Framework (Section 4.4.3)

Experiment-specific settings. A grid search was conducted over nine (α, β) formula combinations: *a* ∈ {0.02, 0.10, 0.20} and *b* ∈ {0.05, 0.15, 0.40}. For each combination, the Simulation model (Section S1.7) was re-executed on the full cohort. A 100-trial Bayesian TPE optimization (20 random startup trials) was run per combination, jointly optimizing four hyperparameters: TMAE ρ ∈ [0.60, 0.90], TMAE τ ∈ [0.50, 0.80], Radiomics k ∈ {25, 50, 75, 100}, and Clinical retention πC ∈ [0.30, 1.00]. The SM arm was fixed at two outputs (growth rate and necrosis ratio) without selection. The Ensemble model weighted CoxPH and Neural Cox equally (gradient boosting weight = 0). All nine combinations were processed in parallel with checkpoint-and-resume support.

## References

Uno, H., Cai, T., Pencina, M. J., D’Agostino, R. B., and Wei, L. J. (2011). On the C-statistics for evaluating overall adequacy of risk prediction procedures with censored survival data. Statistics in Medicine 30, 1105–1117.

Heagerty, P. J., Lumley, T., and Pepe, M. S. (2000). Time-dependent ROC curves for censored survival data and a diagnostic marker. Biometrics 56, 337–344.
